# Vulnerability of the foot's morphological structure to deformities caused by foot loading paradigm in school-aged children: a cross-sectional study

**DOI:** 10.1038/s41598-021-82475-y

**Published:** 2021-02-02

**Authors:** Beata Szczepanowska-Wolowiec, Paulina Sztandera, Ireneusz Kotela, Marek Zak

**Affiliations:** 1 Institute of Health Sciences, Collegium Medicum, The Jan Kochanowski University, 25-317 Kielce, Poland; 2 Rehabilitation Clinic, Provincial General Hospital, 25-310 Kielce, Poland; 3 Institute of Medical Sciences, Collegium Medicum, The Jan Kochanowski University, 25-317 Kielce, Poland; 4 Central Clinical Hospital of the MSWiA, 02-507 Warsaw, Poland

**Keywords:** Population screening, Health care, Paediatrics, Public health

## Abstract

The study aimed to assess the association between the key predictive foot structure variables and its loading paradigm in 625 school-aged children. Clinical appraisal relied primarily on having the plantar parts of their feet comprehensively assessed with Podoscan 2D Foot CAD, and a dynamometer platform, the research tools of choice widely acknowledged for their overall accuracy and reliability, with a view to determining the distribution of respective foot loads, as well as addressing both balance and gait issues. The Clarke's angle, Wejsflog index, length and width of the feet, regardless of gender, proved the key predictive variables for the foot-loading paradigm. Notably the Clarke's angle, construed the most sensitive variable in assessing flat-footedness, offered an extra added value in overall investigative effort. The actual design of the study protocol effectively complements a standard clinical assessment procedure, whereas by comprehensively addressing those variables, it is also believed to aid clinicians in gaining an extra, hands-on, diagnostic potential, so that any teenagers exposed to the highest risk of developing foot deformities could effectively be identified through pertinent screening tests, and consequently offered a task-oriented, therapeutic management, specifically aimed at preventing potential postural complaints in later life.

## Introduction

Back in the 15th c., Leonardo da Vinci, whilst expressing his appreciation for the phenomenon of the human foot, wrote: "The human foot is a machine of masterful construction and a work of art". This "masterful construction" boasts a fundamental role in a human locomotor system, as its correct anatomical structure actually determines performance and efficiency of its supporting, carrying, cushioning, transporting, and stabilizing functions. Hence, any dysfunctions within the foot may appreciably impact overall biomechanics of the entire body^1^.

Musculoskeletal system in a child, still remaining at its development stage, is quite vulnerable to a number of both inherent and environmental factors which predispose to acquiring postural defects. Foot deformities acquired at that vulnerable stage may well cause a variety of functional problems, or even a fully-fledged disability in adult life^2,3^. Foot deformities alter a foot's pressure (foot load) on the ground, and consequently induce changes in other parts of the locomotor system through the so called knock-on effect^2–4^. The consequences of foot deformities and defective foot loading paradigms may potentially affect not only the lower limbs, but also the entire postural system in an individual^5^.

There are several methods of assessing the variables of the plantar part of the foot, some of them more complex than others, e.g. Chippaux-Smirak Index, Staheli Index, Hernández-Corvo Index, Clarke's angle^6–9^. This said, there are also numerous reports attesting to overall credibility and reliability of determining the median longitudinal arch, whilst making use of the Clarke's angle^9–13^. Some investigators even demonstrated there were no differences whatsoever between respective measurement methods, even though they appreciably differed in complexity and ease of application.

Since clinical studies focused on how the foot deformities sustained in their teens may specifically affect overall postural stability in school-aged children are generally scarce, the Authors aimed to address this deficit by gaining specific insights into prevalent associations between the morphological structure of the foot (notably its arching) and the actual load distribution in its plantar part (i.e. its loading paradigm).

Considering that specific types of foot deformities sustained by children are hypothesised to be the actual predisposing factors in potentially bringing on a cascade of degenerative changes throughout the body, subsequently translating into serious postural defects and complaints in later life, their effective identification was pivotal to the entire investigative effort.

The research tools of choice (primarily Podoscan 2D Foot CAD) had deliberately been opted for as the optimal ones for the purpose. They are perfectly suited for an easy, practical, on-site application in any settings, be that in clinical or outpatient facilities, or in a diversity of non-medical settings, e.g. schools, i.e. yet another unbeatable advantage in terms of effectively handling complex logistics of any field research.

As the design of the study protocol actually complements a standard clinical assessment paradigm, it is also meant to offer an extra, hands-on, long-term diagnostic potential for clinicians and physiotherapists alike, possibly with a view to comprehensively aiding nationwide preventive policies.

## Results

The survey involved 625 children (327 boys and 298 girls) aged 10–15 years, randomly selected from primary schools, representing both an urban and rural environment of a single province, whose characteristics are presented in Table 1. All study subjects had been granted consent to participate in the study protocol by their parents/guardians.

**Table 1:** Basic characteristics of the study subjects.

Variable	Boys (n = 327) Mean ± SD	Girls (n = 298) Mean ± SD	Z	p
Body mass [kg]	45.29 ± 13.18	43.89 ± 11.5	− 0.743	0.458
Height [cm]	152 ± 0.12	151 ± 0.1	− 0.294	0.769
BMI	19.24 ± 3.72	18.85 ± 3.34	− 0.964	0.335

Mean—arithmetic mean, SD—standard deviation, Z—statistical values of the Mann–Whitney–Wilcoxon test for two independent samples, BMI- body mass index, p-significance level.

Being well aware that while making use of the percentiles, it is also worthwhile to refer to the studies focused on specific populations which take into account the effect of external and environmental factors characteristic of a given nationality, the Authors made use of the BMI charts developed by Kułaga et al.^14^, based on the studies of 22,211 Polish children.

There were no statistically significant differences in body weight (p = 0.458), body height (p = 0.769) and BMI (p = 0.335) in the subgroups stratified by gender.

**Figure 1:**
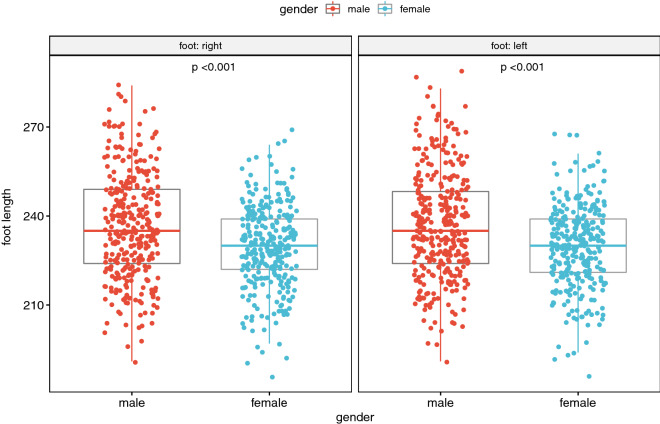
Dependence of foot length on gender^15,16^.

**Figure 2:**
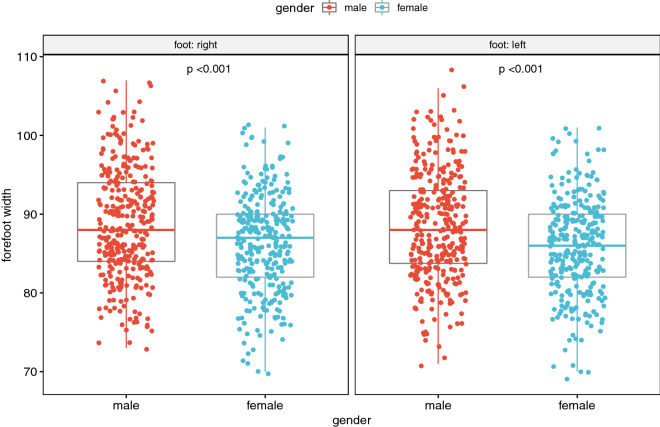
Dependence of forefoot width on gender^15,16^.

**Figure 3:**
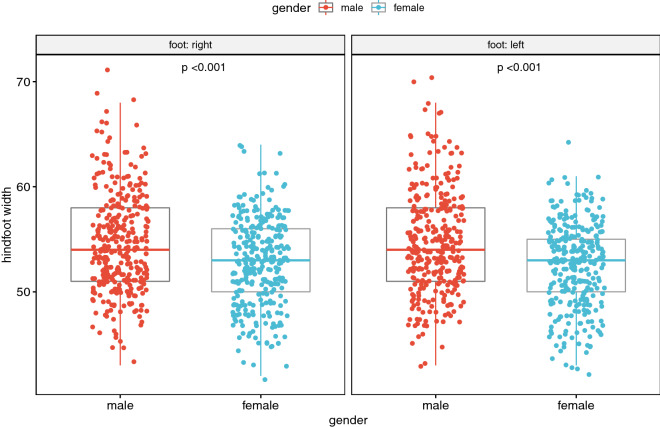
Dependence of hindfoot width on gender^15,16^.

**Figure 4:**
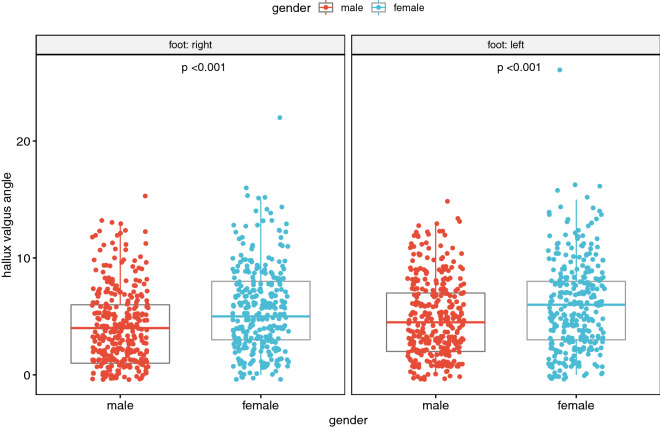
Dependence of the hallux valgus angle on gender^15,16^.

The following figures indicate statistically significant relationships between the morphological variables parameters of the subjects' feet and gender (i.e. Fig. 1, 2, 3, 4). The Mann–Whitney–Wilcoxon test was applied to determine the significance of the two independent results under study.

Table 2 indicates statistically significant differences in the foot load variables between girls and boys.

**Table 2:**
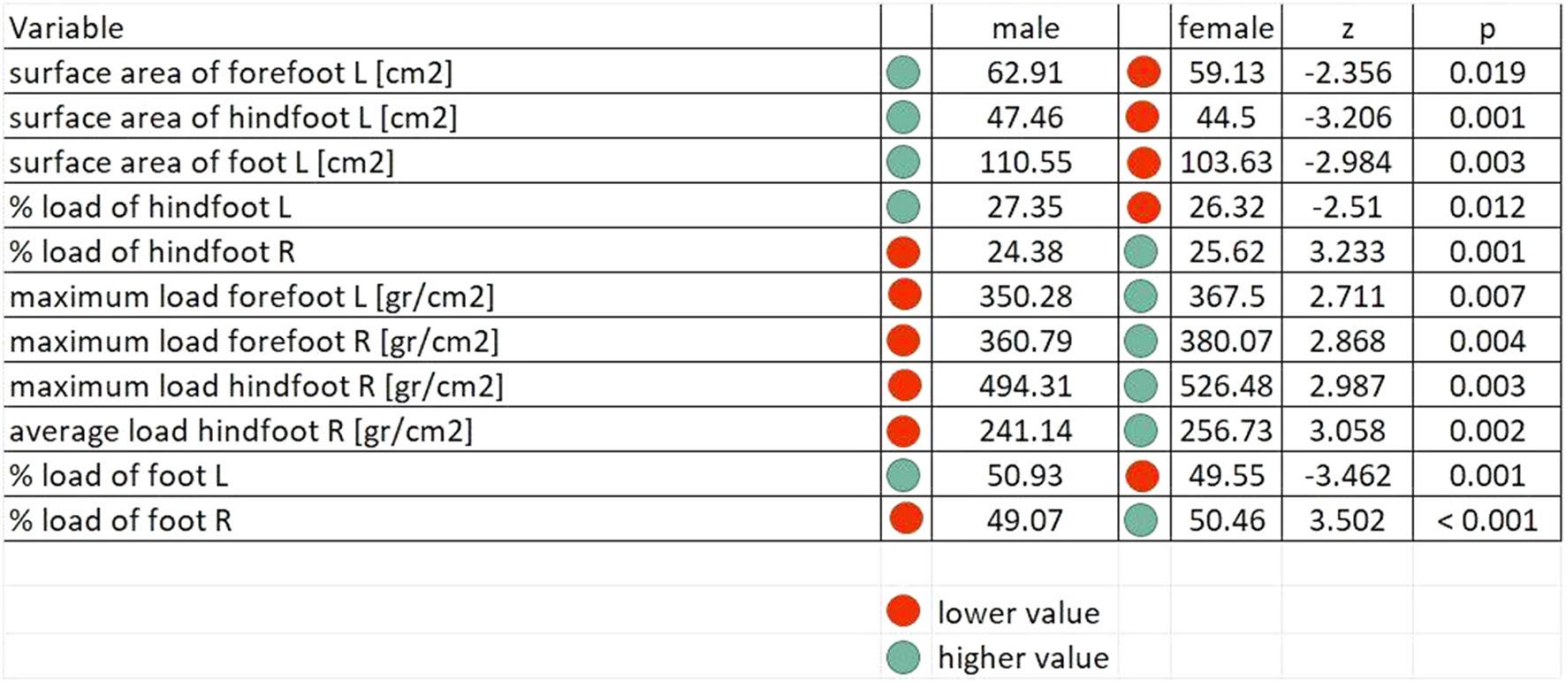
Average values of the foot load variables, stratified by gender.

Z—value of the Mann–Whitney-Wilcoxon test statistics for two independent results, p—level of significance.

In Tables 2 and 3, Spearman's rank correlation ρ was applied to determine the relationship between forefoot and hindfoot load and the morphological characteristics of the feet. Statistically insignificant correlations were colour-coded.

**Table 3:** Associations between the forefoot load and morphological features of the feet.

Variables	Surface area in cm^2^	Load in %	R/F weight ratio %
Clarke’s angle L	ρ = − 0.187 p < 0.001	ρ = − 0.137 p = 0.001	ρ = − 0.172 p < 0.001
Clarke’s angle R	ρ = − 0.235 p < 0.001	ρ = − 0.177 p < 0.001	ρ = − 0.164 p < 0.001
Wejsflog index L	ρ = − 0.267 p < 0.001	ρ = − 0.149 p < 0.001	ρ = − 0.145 p < 0.001
Wejsflog index R	ρ = − 0.279 p < 0.001	ρ = − 0.117 p = 0.003	ρ = − 0.149 p < 0.001
Hallux valgus angle α L	ρ = 0.007 p = 0.863	ρ = 0.050 p = 0.213	ρ = 0.119 p = 0.003
The fifth toe angle β L	ρ = 0.284 p < 0.001	ρ = 0.178 p < 0.001	ρ = 0.177 p < 0.001
The fifth toe angle β R	ρ = 0.314 p < 0.001	ρ = 0.179 p < 0.001	ρ = 0.204 p < 0.001

Out of the morphological features of the girls' feet, the Clarke's angle of the right foot was the strongest determinant of the forefoot and hindfoot load (%), and R/F weight ratio (%) of the forefoot and hindfoot. In the case of an average load on the forefoot (gr/cm^2^), regardless of whether the right or left foot, the most strongly linked variable proved the length of the right foot, while the average load on the hindfoot (gr/cm^2^) was most strongly determined by its actual width, whose load was the variable under study Table 4.

**Table 4:** Associations between the hindfoot load and morphological features of the feet.

Variables	Surface area in cm^2^	Load in %	R/F weight ratio %
Clarke’s angle L	ρ = − 0.074 p = 0.063	ρ = 0.176 p < 0.001	ρ = 0.172 p < 0.001
Clarke’s angle R	ρ = − 0.129 p = 0.001	ρ = 0.126 p = 0.002	ρ = 0.165 p < 0.001
Wejsflog index L	ρ = − 0.186 p < 0.001	ρ = 0.093 p = 0.020	ρ = 0.145 p < 0.001
Wejsflog index R	ρ = − 0.210 p < 0.001	ρ = 0.121 p = 0.002	ρ = 0.150 p < 0.001
Hallux valgus angle α L	ρ = − 0.039 p = 0.330	ρ = − 0.155 p < 0.001	ρ = − 0.119 p = 0.003
The fifth toe angle β L	ρ = 0.210 p < 0.001	ρ = − 0.131 p = 0.001	ρ = − 0.177 p < 0.001
The fifth toe angle β R	ρ = 0.235 p < 0.001	ρ = − 0.163 p < 0.001	ρ = − 0.204 p < 0.001

In the group of boys a set of similarly strong predictors were encountered. The load (%) of the forefoot and the right hindfoot were the variables accounting for the differences, though. The most important dependent variable was the angle of the 5th toe β of the left foot, whereas in the case of the average load of the forefoot (gr/cm^2^), the most important dependent variable, was the actual width of the forefoot of the right foot.

More details on the stepwise regression models applied may be found in Tables 5 and 6, further below. They comprise all pertinent predictors, ranked from most important to the least important ones, in conjunction with the Fisher-Snedecor's F statistic values, and the determination factor of the entire model—R2. The value of R2 corresponds to the part of the variance accounted for by the model. The closer the value approaches 1, the better the model.

**Table 5:** Stepwise regression models in the group of girls.

Dependent variable	Predictor	F	p	R^2^
% load of L forefoot	Clarke’s angle R	14.03	< 0.001	0.1495
	Wejsflog index L	12.46	< 0.001	
	Foot length R	4.15	0.043	
	Hindfoot width R	20.68	< 0.001	
% load of R forefoot	Clarke’s angle R	11.31	< 0.001	0.1348
	The fifth toe angle β R	8.18	0.005	
	Hindfoot width L	5.50	0.02	
	Forefoot width R	20.51	< 0.001	
R/F weight ratio % L forefoot	Clarke’s angle R	19.03	< 0.001	0.1972
	The fifth toe angle β R	15.94	< 0.001	
	Forefoot width L	4.59	0.033	
	Hindfoot width L	32.19	< 0.001	
R/F weight ratio % R forefoot	Clarke’s angle R	12.74	< 0.001	0.1394
	The fifth toe angle β R	10.23	0.002	
	Hindfoot width R	5.53	0.019	
	Forefoot width R	18.83	< 0.001	
Average load of the L forefoot gr/cm^2^ log	Foot length R	61.84	< 0.001	0.2303
	Hallux valgus angle α R	9.16	0.0026	
	Wejsflog index R	6.80	0.0095	
	The fifth toe angle β R	4.71	0.0307	
	Hallux valgus angle α L	4.29	0.0392	
Average load of the R forefoot gr/cm^2^ log	Foot length R	76.42	< 0.001	0.2274
	Wejsflog index R	5.35	0.0213	
	The fifth toe angle β R	4.20	0.0412	
Average load of the L hindfoot gr/cm^2^	Hindfoot width L	29.18	< 0.001	0.1382
	The fifth toe angle β R	8.69	0.0034	
	Clarke’s angle R	8.96	0.0029	
Average load of the R hindfoot gr/cm^2^	Hindfoot width R	29.05	< 0.001	0.1161
	Clarke’s angle R	4.50	0.0348	
	The fifth toe angle β R	4.84	0.0285	

F—value of the Fisher-Snedecor test statistics, R^2^—coefficient of determination, log- logarithm of the variable under study.

**Table 6:** Stepwise regression models in the group of boys.

Dependent variable	Predictor	F	p	R^2^
% load of L forefoot	Clarke’s angle R	18.57	< 0.001	0.1977
	The fifth toe angle β L	4.67	0.031	
	The fifth toe angle β R	20.42	< 0.001	
	Hindfoot width R	5.41	0.021	
	Forefoot width R	29.59	< 0.001	
% load of R forefoot	The fifth toe angle β L	17.77	< 0.001	0.1233
	Clarke’s angle R	10.79	0.001	
	Hindfoot width R	4.91	0.027	
	Forefoot width R	11.54	< 0.001	
R/F weight ratio % L forefoot	Clarke’s angle R	25.52	< 0.001	0.1281
	The fifth toe angle β R	21.81	< 0.001	
R/F weight ratio % R forefoot	Clarke’s angle R	11.61	< 0.001	0.1874
	The fifth toe angle β L	26.85	< 0.001	
	Hindfoot width R	11.35	< 0.001	
	Forefoot width R	23.99	< 0.001	
Average load of the L forefoot gr/cm^2^ log	Forefoot width R	68.35	< 0.001	0.1746
Average load of the R forefoot gr/cm^2^ log	Forefoot width R	63.00	< 0.001	0.1847
	Wejsflog index R	4.39	0.0369	
	Clarke’s angle L	5.34	0.0214	
Average load of the L hindfoot gr/cm^2^	Hindfoot width L	19.97	< 0.001	0.1280
	The fifth toe angle β R	8.10	0.0047	
	Clarke’s angle R	19.10	< 0.001	
Average load of the R hindfoot gr/cm^2^	Hindfoot width R	36.60	< 0.001	0.1647
	Clarke’s angle R	13.82	< 0.001	
	The fifth toe angle β R	12.88	< 0.001	

## Discussion

The results yielded by the present study corroborate prevalent association established between the morphological structure of the foot (notably its arching) and the actual load distribution in its plantar part (i.e. loading paradigm) in the school-aged children.

To date, any academic research endeavours making use of objective assessment tools have primarily been focused on adults, so consequently there is an appreciable scarcity of wider scoped studies assessing these associations among the school-aged children and adolescents, which prompted the Authors to address this particular deficit by gaining some specific insights into such dysfunctions in the youngsters, with a view to juxtaposing the findings against the background of published research on adults.

Apart from their strictly academic aspect, the present findings may well become instrumental in appreciably enhancing a scope of diagnostic procedures applied to date in assessing children's feet. The results effectively highlight close associations between the morphological structure of the foot and its loading paradigm. The results of stepwise regression model allow to have the variables which account for the load factors under study effectively identified.

Appraisal of specific morphological features of the girls' feet indicated that the Clarke's angle of the right foot was the strongest predictor of the loads borne by both the forefoot and the hindfoot, and the R/F weight ratio. Publications on the subject comprise reports on a dimorphism of longitudinal foot structure. Female feet boast better arched longitudinal vaulting, which corroborates the assertion based in our own research, whereby the Clarke's angle is deemed the most important predictive variable among them all.

It might well be assumed that the essential predictors regarding a foot loading paradigm pertain to the right foot. This may well be attributable to the fact that one leg is usually dominant in every individual, mostly the right one. The Clarke's angle takes into account the following variables, i.e. R and L forefoot load, L and R forefoot weight factor, and mid-foot load L and R. It might therefore be inferred that the right limb boasting a manipulative function demonstrates a readiness for undertaking defensive reactions. A similar association was asserted by Puszczalowska-Lizis et al. in her own study^12^.

Lee et al.^17^, whilst evaluating the FPI and its correlation with flat-footedness in children, noted a significant association. Chang et al.^4^ also established the relationship between the foot arches and the foot load whilst walking. Fernández-Seguín et al.^18^, when assessing the foot pressure between a normal and cavus foot in adults, noticed the differences in the cavus foot load. A similar association was observed in our own research. The Clarke's angle values, which describe the vaulting of the foot's longitudinal arch (flat, normal, cavus foot), demonstrated there was an essential variable which accounted for the foot loading paradigm, both among the girls and boys. Pauk et al.^19^ also indicate the sensitivity and reliability of the Clarke's angle, both as a quantitative variable, and as a variable useful in determining the pressure of the feet with regard to flat-footedness.

A vital factor affecting overall correctness of load distribution is the actual positioning of the toes, especially of the first and the fifth toe. Hallux valgus and the fifth toe β are responsible for even distribution of foot pressure (front support zone). Mickle et al.^2^, Puszczalowska-Lizis et al.^20^, while studying groups of individuals of different age, noted that altering the positioning of the hallux valgus translated into altering of the forefoot pressure. In our own study, we observed the angle of the hallux valgus, and the angle of the varus of the fifth toe β, which were important predictive factors for the foot loading variables, both in girls and boys.

The forefoot width and the Wejsflog index also made essential factors, with the gender differences duly accounted for. Sexual dimorphism in boys is apparent in the tendency to more flaccid positioning of the small toe, which might be resultant from loading the lateral edge of the foot. Step regression models in a group of boys gave us grounds to believe that the angle of the small toe was a variable that accounted for the load variable and weight factor of the forefoot.

Even slight distortions in the forefoot area are instrumental in increasing the pressure of the feet, which may lead to further deformities^5,21,22^. Deformities originating within the foot's anatomical structure initially disrupt its function, but at the following stage the disorder spreads out to the entire structure of the body, and may consequently result in postural complaints. It seems quite prudent, therefore, to have the morphological structure of the feet and the distribution of pressure regularly monitored, as apparent differences in their structure and the manner of loading may subsequently result in many disorders in adulthood. The distribution of foot pressure depends on the person's body weight, but also on any pathological irregularities within its anatomical structure^23^.

The assessment of the foot structure and load distribution is essential in terms of prevention strategies and pertinent remedial measures^17,21,23–30^. Screening tests facilitate regular monitoring of overall condition of the feet, whilst offering a diversity of easy-to-apply, non-invasive, objective, and reliable testing methods. Making use of a podoscan clearly offers such an easy-to-apply, standardized method. When assessing the basic morphological variables of the feet, special attention should be paid to their association with the distribution of the pressure against the ground, i.e. individual foot loading paradigm.

Spotting abnormal distribution of the foot pressure, as encountered in various disorders, would be bound to make the clinicians more receptive to the issue of comprehensive prevention, including extra protection against any deformities, as well as pertaining to overall postural care^31–35^.

Upon noticing any structural abnormalities within the foot area, detailed diagnostic tests are urgently required, so as to identify the exact nature of the problem at issue, and subsequently map out an individually tailored, target-oriented, therapeutic regimen designed in full consideration of the foot biomechanics, as adversely affected by a specific type of abnormality.

It should also be highlighted at this juncture that the very design of the study protocol actually complements a standard clinical assessment paradigm, while offering an extra, hands-on diagnostic potential for clinicians. Consequently, developing an algorithm for an early stage prevention of foot deformities in school-aged children would appreciably contribute to developing a nationwide prevention strategy, primarily with a view to streamlining the allocation of strained public healthcare resources.

## Conclusions

1. The Clarke's angle of the right foot makes an essential predictive factor accounting for the foot loading paradigm in girls and boys alike.
2. Length and width of the foot stand for the predictive variables accounting for the foot loading paradigm in girls and boys alike.
3. In boys, the dependent variable for assessing the foot load % is the angle of the foot's small toe β.
4. The Clarke's angle, Wejsflog index, and the length and width of the foot stand for the predictive factors accounting for the foot loading paradigm.

## Research design and methods

The following inclusion criteria were adopted: informed consent to participate in the study protocol, full documentation of the study, no disorders in the locomotor system, as assessed through an interview. The exclusion criteria were as follows: no informed consent available to participate in the study protocol, incomplete documentation of the study, disorders in the locomotor system, as assessed through an interview, metabolic disorders that might potentially affect the skeletal system^20^.

### Anthropometric measurements

Body weight was assessed using the Tanita weight (Japanese-made, 93/42/EEC Annex II, accuracy ± 0.1 kg), while the height by using the SECA height gauge (German-made, 93/42/EEC, 2007/47/EC, accuracy 0.01 m)^21,35^.

The Tanita scales analyses the body mass composition with the aid of electrical bio-impedance technology. The device is fitted with 8 sensors, placed underneath the platform on which a person stands barefooted, as well as in the handles to be held by the subject during the testing procedure. The measurement of electrical bio-impedance, dependent upon respective electrical conductivity of specific body tissues, facilitates the actual assessment of whether a person happens to be undernourished, obese, or just overweight^35^.

### Morphological structure of the foot and the fool loading paradigm

The Podoscan 2D Foot CAD is an instrument facilitating assessment of the plantar part of the foot in static conditions. Making use of FreeSTEP BASIC software allows to measure, determine respective angles, compare, and subsequently retain the photographs on file. High resolution (1600 DPI) facilitates obtaining accurate measurements of pertinent foot variables, as well as implementing the foot's functional assessment. Pertinent variables, i.e. length, width, angles, and axes of the feet were determined. The subjects stood on the Podoscan barefooted, lower limbs straight, feet parallel to each other, upper limbs positioned along the torso^21,35^.

**Figure 5:**
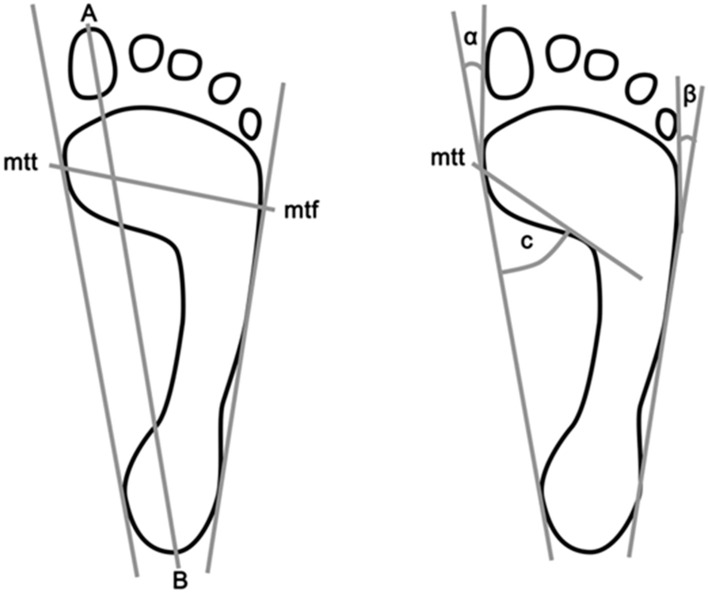
The method of determining the foot variables under study. A-B—foot length, mtt-mtf-foot width, C—Clarke's angle, α—hallux valgus angle, β—varus deformity of the 5th toe. (Source: Own research^21^. “Feet deformities and their close association with postural stability deficits in children aged 10–15 years,“ by Szczepanowska-Wolowiec et al.^21^ Copyright 2019 by the BMC).

The following indicators were assessed:

- foot length—the line connecting the furthest points of the forefoot and hindfoot—in mm
- forefoot width—the line joining the most extreme points on the head of the first (mtt) and the fifth metatarsal bone (mtf)—in mm
- Clarke’s angle—the angle between the tangent of the medial edge of the foot and the line joining the point of the largest indentation and contact of the medial tangent with the foot edge—in °
- the Wejsflog (W) index—length-to-width ratio
- the hallux valgus angle (α)—the angle between the tangent to the medial edge of the foot and the tangent of the edge of the hallux, derived from the mtt point—in °
- The angle of the varus deformity of the fifth toe (β)—the angle between the tangent of the lateral edge of the foot and the tangent of the edge of the fifth toe derived from the point mtf—in ° (Fig. 5)^21,35^.

The value of the Clarke's angle was used to assess the longitudinal arching of the foot. Hence: flat foot < 30º, lowered arching of the foot 31°–41°, normal foot 42°–54°, elevated arching of the foot > 55°.

The Wejsflog index (length/width of the foot) is a variable originating in the assessment of the transverse vaulting. The toe angle α was also assessed—normal value up to 9°. The mean values of the varus deformity of the 5th toe for girls were: Mean-14.43 ± SD 5.06 in the left foot, Mean-14.88 ± SD 5.48 in the right foot, Mean-13.77 ± SD 4.93 for boys in the left foot, Mean-15.2 ± SD 5.08 in the right foot.

A dynamometer platform (FreeMed, Sensor Medica, Italian-made) with FreeStep Pro software was used for stabilometer testing. The actual surface of the platform is 635 mm × 700 mm, whereas the area of 500 mm × 600 mm is fitted out with sensors, so that the distribution of respective feet loads, balance, and gait may effectively be assessed^21,35^.

The subjects stood at ease on the platform, the upper limbs along the torso with their lower limbs parallel to each other. The subjects were asked to stand perfectly still, with the eyes fixed on the point right in front of them.

The following variables of the left and right foot were assessed:

- surface in cm^2^, forefoot and hindfoot
- Load %, forefoot and hindfoot
- weight factor R/F % (coefficient of mass distribution on the forefoot and hindfoot)
- max. load gr/cm^2^, forefoot and hindfoot
- average load gr/cm^2^, forefoot and hindfoot
- total area in cm^2^
- total foot load in %
- total foot load in kg

**Figure 6:**
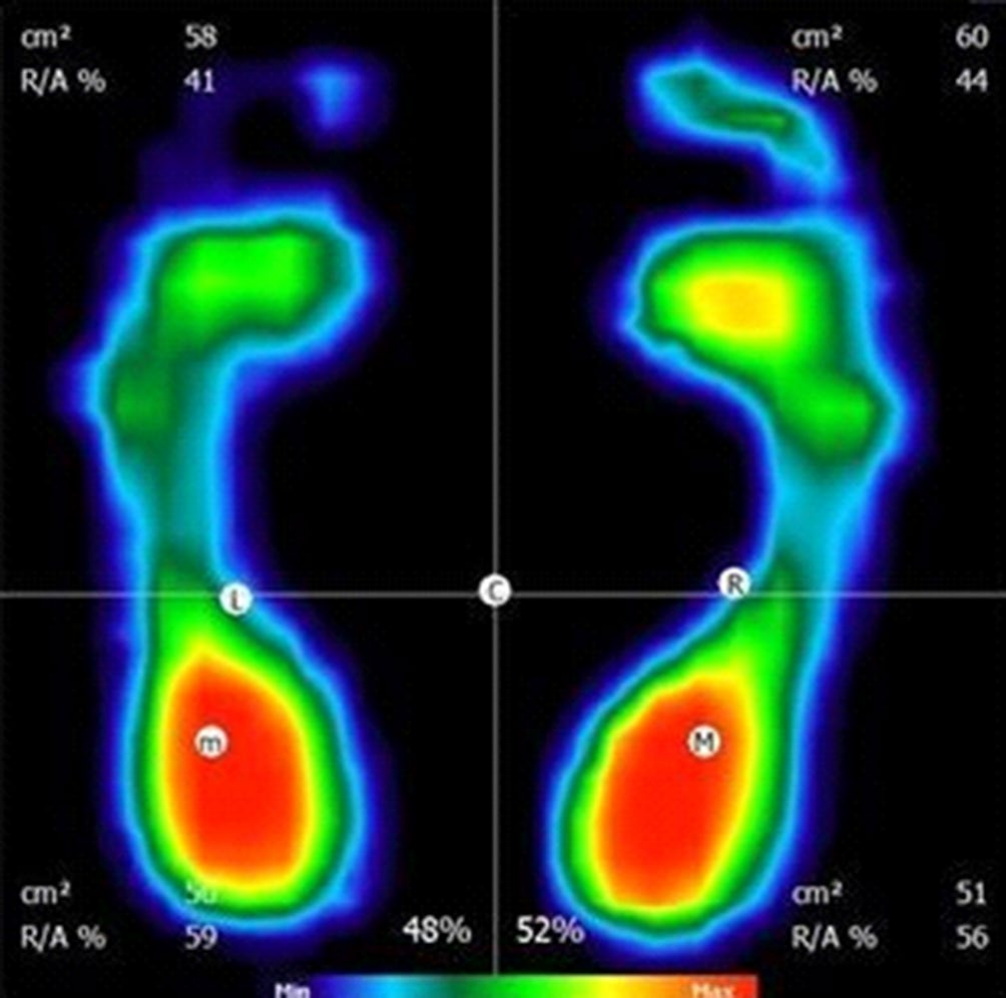
An example of a foot pressure test, as carried out on the FreeMed platform, in static conditions. (Source: Own research).

Figure 6 shows an analysis of the foot pressure on the ground.

### Statistical analysis

The R statistical programme, v. 3.5.0, was used to process the results^16^. Basic measures of descriptive statistics, i.e. arithmetic mean, standard deviation, were applied throughout. The variables of the left and right foot structure, stratified by gender, were assessed while using the Mann–Whitney–Wilcoxon test for two independent results, also in order to assess the relationship between the foot load and gender. Spearman's rank correlation was used to analyse the variables pertaining to the morphological structure of the foot and respective foot loads. Progressive stepwise regression was applied to assess how the foot variables affected the foot load indices.

Prior to the regression analysis, the Shapiro–Wilk test was used to check the normality of the distribution of the dependent variable. Whenever the test indicated the variable did not have a normal distribution, the Box-Cox transformation was applied. The quality of the model was assessed using the R^2^ determination factor. Respective variables were considered statistically significant and consequently included in the model, if the F statistics from the Fisher-Snedecor test had a value of p < 0.05.

### Study limitations

It proved unfeasible to have a group of individuals actively involved in sports effectively identified within the study population. Otherwise, comparison of the respective results/variables under study would convincingly highlight the adverse effects of physical inactivity.

Another essential limitation consisted in there having been no possibility to have the study population stratified by specific age-ranges, so consequently our results may not fully reflect the key characteristics of respective age groups, owing to small sample sizes.

Initially, we assumed that all footwear worn by the study participants was adequately selected and well-fitting. Our hands-on research practice indicated an altogether different relationship to have been the case, as numerous subjects tended to wear either too tight, or too loose-fitting footwear.

### The compliance statement

All research methods comprised in the study protocol were pursued/implemented in full compliance with pertinent guidelines, regulations, and applicable legislation in place.

### The ethics approval statement

The study design and protocol was granted approval and duly endorsed by the Bioethics Review Committee, established in pursuance of pertinent statutory constraints at the Faculty of Medicine and Health Sciences, The Jan Kochanowski University in Kielce, Poland, following rigorous appraisal of the investigators' application for ethics approval, carried out on June 20, 2016 (Ethics Approval Ref. No. 26/2016).

### The informed written consent statement

Written informed consent was obtained from the parents/guardians of the minor study subjects for their attendance in the study protocol. It was based on the detailed information on the actual aims and research methods to be used, having prior been furnished to them by the Authors.
